# Planar Inverted-F Antenna (PIFA) Using Microfluidic Impedance Tuner

**DOI:** 10.3390/s18103176

**Published:** 2018-09-20

**Authors:** Minjae Lee, Sungjoon Lim

**Affiliations:** School of Electrical and Electronics Engineering, College of Engineering, Chung-Ang University, Seoul 156-756, Korea; iamlmj720@gmail.com

**Keywords:** impedance tuner, matching network, microfluidic, planar inverted-F antenna (PIFA)

## Abstract

This paper proposes a microfluidic impedance tuner that is applied to a planar inverted-F antenna (PIFA). The proposed microfluidic impedance tuner is designed while using a simple double-stub and the impedance is changed by tuning the stub length. In this work, the stub length can be tuned by injecting a liquid metal alloy to the microfluidic channels. Initially, the PIFA operates at 900 MHz with impedance matching of 50 Ω. The impedance is mismatched when a hand is placed close to the antenna. The mismatched impedance is matched to 50 Ω by injecting the liquid metal alloy. The antenna is fabricated on the FR-4 substrate, and the impedance tuner is fabricated on polydimethylsiloxane (PDMS). In order to inject the liquid metal alloy, a piezoelectric micropump and microprocessor are used in the measurement. At 900 MHz, the return loss is successfully tuned from 4.69 dB to 18.4 dB when a hand is placed 1 mm above the antenna.

## 1. Introduction

The presence of a mismatched impedance between circuits results in an overall degradation in circuit performance; impedance matching between circuits is therefore the basis of high frequency electronics. Antennas significantly influence the performance of the entire application, as their impedance values vary significantly even in operating environments having small changes. In particular, as parts of the human body get closer to the antenna, its performance suffers [1]. With the advances in the field of wireless communication systems, an increasing number of components are being incorporated in mobile handset design, reducing the space in which complex advanced antennas can be implemented into the handset. To solve this problem, the use of an impedance tuner is one of the representative methods of matching the antenna impedance. Impedance tuners are widely used to match impedances, such as antennas [2], power amplifier (PA) load-pull [3], and noise characterization [4], and so on. Conventional impedance tuners comprise mainly three different techniques, namely the single-stub [5], double-stub [6], and optimization methods [7]. Impedance tuning has been widely studied using active and passive components, such as microelectromechanical systems (MEMS) components or diode varactors [6,8]. While circuits with many switching elements can have many tuning points, tuning is obtained at the expense of higher cost and insertion loss.

Microfluidic technology has been developed to carry out analysis, screening, and detection of very small quantities of biomaterial and chemical samples. It can be implemented at a less complexity and low cost. Especially, liquid metal in microfluidic channels have been studied for flexible or reconfigurable radio frequency (RF) components [9], such as filters [10], sensors [11,12], absorbers [13,14], antennas [15], and MEMS switch [16].

In this paper, we propose a microfluidic impedance tuner while using liquid metal as a switch. When compared to diode switches, the liquid metal switch provides advantages, such as wider switching range, high power capability, no bias network, and direct current (DC) power consumption, and less parasitic effect. Previously, a microfluidic impedance tuner with a double stub structure has been proposed [17,18] where the liquid metal is continuously injected into long fluidic channels. In this work, the liquid metal is used as a switch in short fluidic channels. Therefore, the injection and extraction are faster, easier, and reliable. In addition, the injection and extraction of liquid metal are controlled a microcontroller and micropump for practical implementation. Finally, the proposed microfluidic impedance tuner is tested with a planar inverted-F antenna (PIFA).

The proposed microfluidic impedance tuner is designed using a simple double-stub and the impedance is changed by tuning the stub length. The stub length can be tuned by injecting a liquid metal alloy to the microfluidic channels. The PIFA with the proposed impedance tuner is designed while using circuit and full-wave simulation. Its performance is numerically and experimentally demonstrated. The design and fabrication processes are explained.

## 2. Design of PIFA and Microfluidic Impedance Tuner

### 2.1. PIFA Design

**Figure 1:**
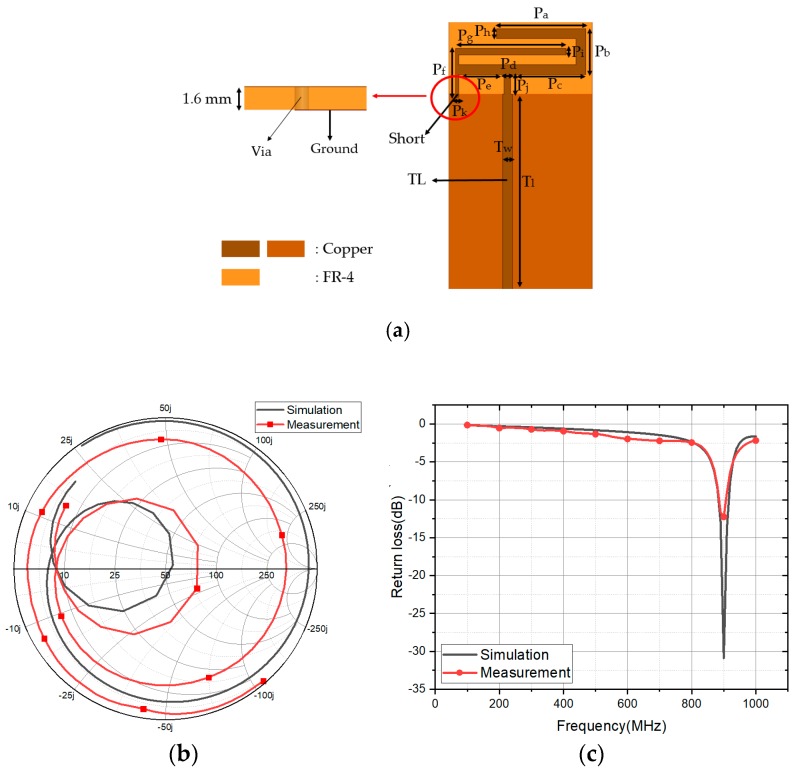
(**a**) Geometrical parameters of planar inverted-F antenna (PIFA); (**b**) Simulated and measured input impedances on Smith Chart; and, (**c**) Simulated and measured return losses.

First of all, the conventional PIFA is designed to resonate at 900 MHz using ANSYS high frequency structure simulator (HFSS), as shown in Figure 1 [19,20]. The FR-4 board (size: 44 × 82 mm^2^, thickness: 1.6 mm) is used as the antenna substrate. Its permittivity and loss tangent are 4.2 and 0.02, respectively. The geometrical parameters are indicated in Figure 1a as P_a_ = 28.8, P_b_ = 14, P_c_ = 23, P_d_ = 2, P_e_ = 14, P_f_ = 15, P_g_ = 34, P_h_ = 2, P_j_ = 6, P_k_ = 1, T_w_ = 3, and T_l_ = 60 mm. Its simulated measured impedances are plotted on Smith Chart, as shown in Figure 1b,c shows the simulated and measured return loses. At 900 MHz, the simulated and measured returns losses are −30.88 dB and −12.25 dB, respectively.

### 2.2. Impedance Measurement at Touched and Untouched States

**Figure 2:**
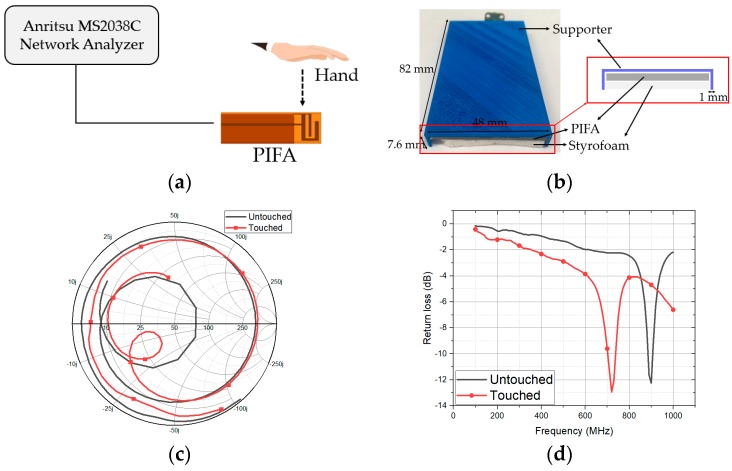
(**a**) Measurement setup of PIFA at touched and untouched states; (**b**) Photograph of the 3D printed mockup with PIFA; (**c**) measured input impedances on Smith Chart at touched and untouched states; (**d**) measured reflection coefficients at touched and untouched states.

In order to design the impedance tuner, we measured input impedance of the PIFA under two scenarios of touched and untouched cases, as illustrated in Figure 2a. It is expected that the impedance is mismatched when the PIFA is touched by a hand. Before measuring the impedance, we fabricate the mockup using a three-dimensional (3D) printer with poly lactic acid (PLA) filament. As shown in Figure 2b, the size of the mockup is 48 × 82 × 7.6 mm^3^ and thickness is 1 mm. The Styrofoam is inserted to fix the antenna position with the SubMiniature version A (SMA) connector. Figure 2c,d show the measured input impedance and return loss of the PIFA with the mockup for touched and untouched states, respectively. At 900 MHz, the return loss of the untouched state is 12.25 dB. When the top of the mockup is touched by a hand, the return loss becomes 5.946 dB at 900 MHz. The measured impedances will be used for a microfluidic impedance tuner design.

### 2.3. Microfluidic Impedance Tuner Design

**Figure 3:**
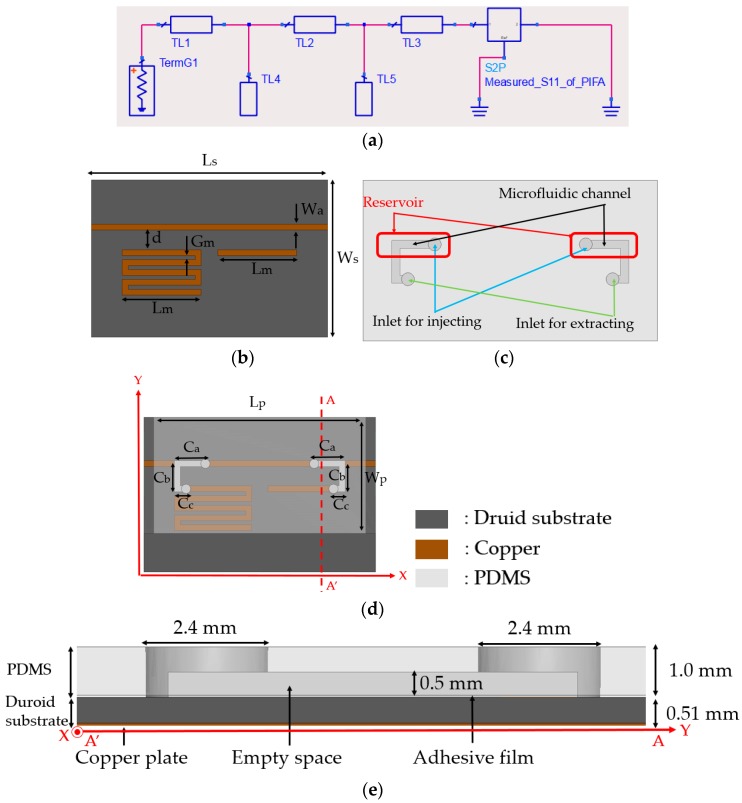
(**a**) Measurement setup of PIFA at touched and untouched states; (**b**) Photograph of the 3D printed mockup with PIFA; (**c**) measured input impedances on Smith Chart at touched and untouched states; (**d**) measured reflection coefficients at touched and untouched states; and, (**e**) Side view of the proposed microfluidic impedance tuner.

In order to design the microfluidic impedance tuner, Keysight Advanced Design System (ADS) circuit simulator is used. As shown in Figure 3a, the double-stub matching network is designed by using the measured impedance of the PIFA at the touched state. At the untouched state, the 50-Ω transmission line is not connected to both two open stubs (TL4 and TL5). In order to connect to stubs, liquid metal is used. Electrical length of TL1, TL2, and TL3 are 0.02λ, 0.125λ, and 0.02λ, respectively. In addition, electrical length of TL4 and TL5 are 3.27λ and 0.75λ, respectively. Characteristic impedance of all transmission lines is 50-Ω. Based on the ADS design, the impedance tuner is designed in a microstrip line using ANSYS HFSS. Figure 3b shows the layout of the proposed impedance tuner where two open stubs are not connected to the 50-Ω microstrip line. In order to minimize the space, the TL4 is realized while using a meander line. The TL5 is not shown because it will be realized using liquid metal. Figure 3c shows the microfluidic channel which is working as a switch. When the channel is filled with liquid metal alloy, two open stubs are connected to the 50-Ω microstrip line. Figure 3d shows the integrated microfluidic impedance tuner. The geometrical parameters of Figure 3b,c are L_s_ = 60, W_s_ = 40, W_a_ = 1.51, d = 5, G_m_ = 1, L_m_ = 20, C_a_ = 7, C_b_ = 8, C_c_ = 3, L_p_ = 55, and W_p_ = 30 mm. The overlap area between the microfluidic channel and the microstrip lines are used as a reservoir for the liquid metal alloy, as illustrated in Figure 3d.

**Figure 4:**
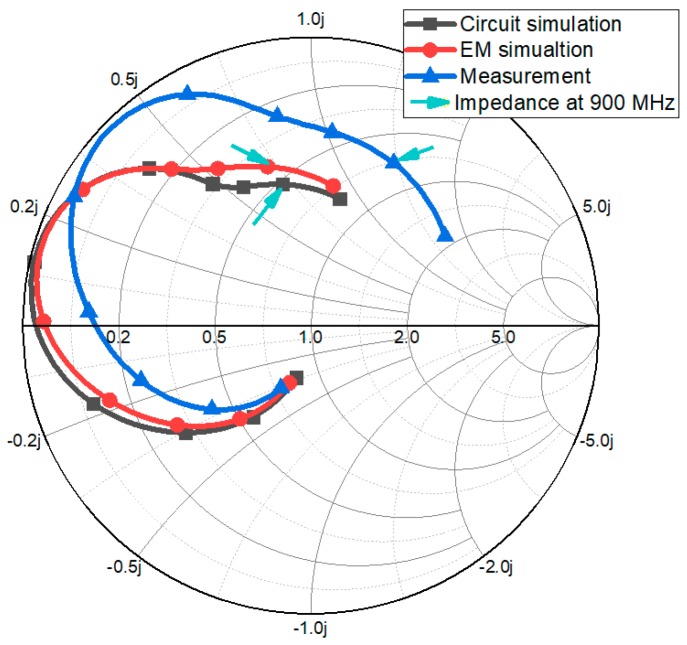
Simulated and measured impedances of the proposed impedance tuner from 100 MHz to 1000 MHz.

The side view of the proposed microfluidic impedance tuner is shown in Figure 3e. The microstrip lines of Figure 3b is realized on a Rogers RT/Duroid 5880 (thickness: 0.51 mm). The microfluidic channels of Figure 3c is realized on polydimethylsiloxane (PDMS). Two substrates are bonded using the adhesive film with a thickness of 0.05 mm. In order to connect a tube to the microfluidic channel, the diameter of the inlet hole is set to 1.2 mm. The tubes will be connected to the micropump. Figure 4 shows the simulated and measured impedances of the proposed impedance tuner at 900 MHz.

## 3. Fabrication and Measurement Result

### 3.1. Fabrication of Microfluidic Channel

**Figure 5:**
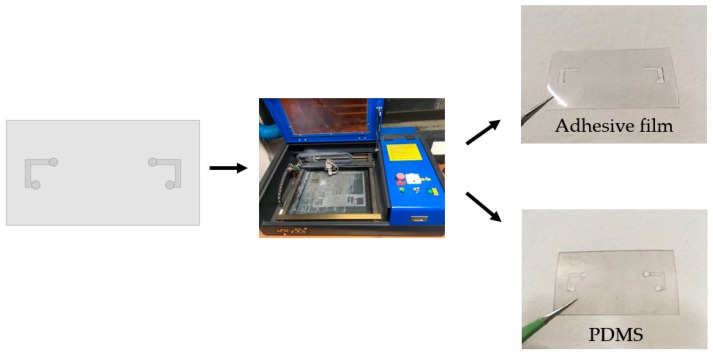
Fabrication process of the polydimethylsiloxane (PDMS) and adhesive film.

Figure 5 shows the fabrication process of the microfluidic channel. The microfluidic channel is fabricated inside a PDMS (Shielding Solutions Ltd., Braintree, UK) while using laser etching. The adhesive film (Adhesives Research, Glen Rock, PA, USA) is also laser etched as the same pattern with PDMS in order to contact the liquid metal on the copper pattern of Duroid substrate. The surface of the liquid metal can be solidified when exposed to the air/oxygen. Because these oxide layers tend to remain to the microfluidic channel as residue, this oxidation problem must be solved for repeatable uses. In this work, in order to solve the oxidation problem, the microfluidic channel is pre-treated with Nafion solution. When hydrochloric acid (HCL) solution is poured, Nafion absorbs the HCL solution. Thus, the oxidation problem can be solved by pushing off the liquid metal in the channel as the residue.

### 3.2. Microcontroller and Micropump

**Figure 6:**
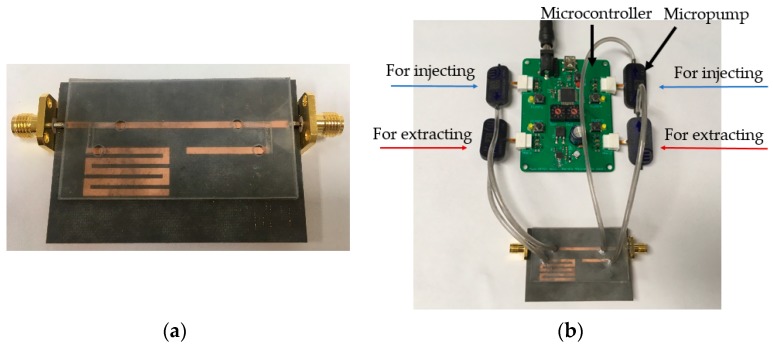
(**a**) Photograph of sample microfluidic impedance tuner; and, (**b**) with the microcontroller and micropump.

Before injecting the liquid metal alloy, the fabricated impedance tuner sample is shown in Figure 6a. EGaIn (Eutectic gallium–indium) is used as the liquid metal alloy. In order to inject and extract the liquid metal alloy into the microfluidic channels, an Mp6 micropump and MP6-QuadEVA microcontroller (Bartels Mikrotechnik GmbH, Dortmund, Germany) are used, as shown in Figure 6b. Because a micropump can pass though only one way, two micropumps are required for injecting and extracting ways of each microfluidic channel. In addition, the pump performance drops when the liquid metal alloy is passed through this pump. Therefore, we used four pumps to prevent the liquid metal alloy from passing through the pump. Finally, total four micropumps are used in this work. Each micropump is connected to the microcontroller with tubes.

### 3.3. Measurement Result

**Figure 7:**
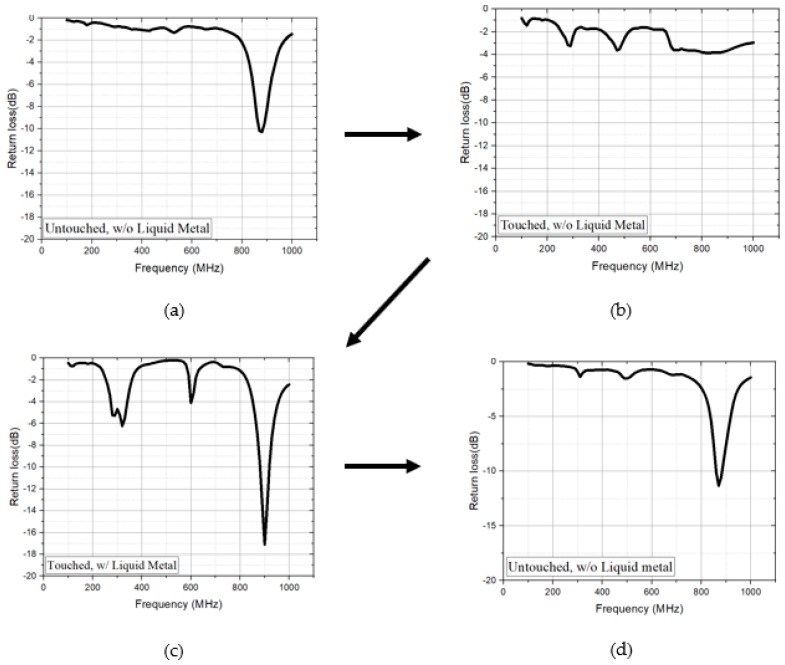
Measurement results of the full prototype with the PIFA and microfluidic tuner in the mockup: (**a**) not touched mockup, and not injecting liquid metal alloy; (**b**) touched mockup, and not injecting liquid metal alloy; (**c**) touched mockup, and injecting liquid metal alloy; and, (**d**) not touched mockup, and extracting liquid metal alloy.

In order to experimentally demonstrate the proposed idea, the microfluidic impedance tuner is connected to the PIFA with the mockup. We measured the S-parameters of the full prototype with the PIFA in the mockup and the microfluidic impedance tuner with the micropumps and microprocessor in four steps. As a first step, the impedance of the full prototype is measured when the mockup is not touched, and liquid metal alloy is not injected. The measured return loss at 880 MHz is 10.288 dB, as shown in Figure 7a. As a second step, the impedance of the full prototype is measured when the mockup is touched, and liquid metal alloy is not injected. The measured return loss at 880 MHz is 3.784 dB, as shown in Figure 7b. As a third step, the impedance of the full prototype is measured when the mockup is touched, and liquid metal alloy is injected. The measured return loss at 880 MHz is 17.104 dB, as shown in Figure 7c. As a fourth step, the impedance of the full prototype is measured when the mockup is untouched and liquid metal alloy is extracted. The measured return loss at 880 MHz is 11.333 dB, as shown in Figure 7d.

### 3.4. Instill Rate

**Figure 8:**
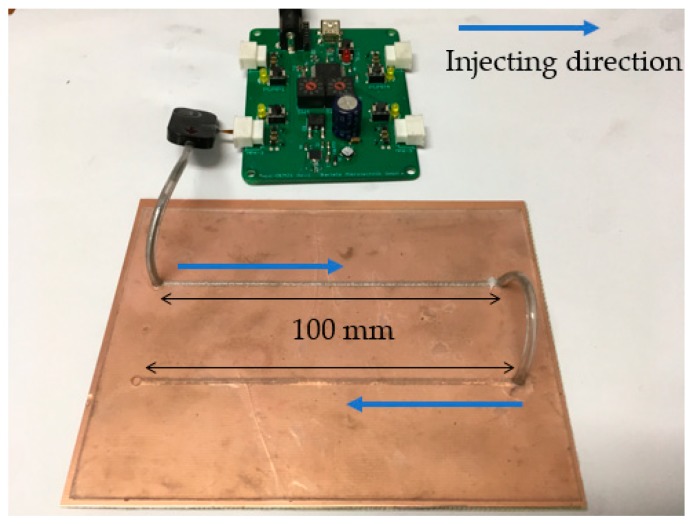
Test setup to measure instill rate of the proposed microfluidic channel with the micropumps and microprocessor.

Although fluidically tuning capability provides merits of less complexity and no DC bias network, low tuning speed can limit its applications. Therefore, it is meaningful to discuss the injection speed for the microfluidic channel. In order to measure the injection speed, we measured the instill rate, which is an indicator of how quickly the proposed impedance tuner can respond. Figure 8 shows the measurement setup. The liquid metal alloy is injected to the microfluidic channel with a 100-mm length and a 0.51-mm width. It takes 1–1.5 s to completely fill the liquid metal alloy into the microfluidic channel. Therefore, the instill rate is 66.7–100 mm/s.

## 4. Conclusions

In this work, we proposed a PIFA with the microfluidic impedance tuner. The proposed impedance tuner is based on double-stub matching circuit and microfluidic channel. The microfluidic impedance tuner is designed while using Keysight ADS and ANSYS HFSS from measured S-parameters of the PIFA with the mockup when the PIFA with the mockup is touched and untouched. When the PIFA is touched by a hand, the mismatched impedance is matched to 50-Ω by injecting the liquid metal alloy to the microfluidic impedance tuner. Therefore, the return losses at 880 MHz can be kept as 10.288 dB and 17.104 dB for the untouched and touched states, respectively. The proposed prototype is successfully tested by using the micropumps and microprocessor. The instill rate of the micropumps and microprocessor is measured as 66.7–100 mm/s with the microfluidic channel. Although the microfluidic switch shows slower switching speed than semiconductor switches, it is useful for applications not requiring fast switching speed. Therefore, the proposed microfluidic impedance tuner can be potentially used for the antenna impedance tuner and millimeter-wave tuning components with more advanced microfluidic technology.
